# Molecular identification of zoonotic taeniids metacestodes in several rodent species trapped in Central Europe

**DOI:** 10.3389/fvets.2025.1571082

**Published:** 2025-05-19

**Authors:** Tomáš Husák, Zuzana Čadková, Ondřej Máca, Marek Kouba, Zdeňka Klimková, Richard Sehnal, Jana Nápravníková, Václava Hrabětová, Ivana Jankovská, Jaroslav Vadlejch, Iva Langrová

**Affiliations:** ^1^Department of Zoology and Fisheries, Faculty of Agrobiology, Food and Natural Resources, Czech University of Life Sciences Prague, Prague, Czechia; ^2^Department of Pathology and Parasitology, State Veterinary Institute Prague, Prague, Czechia; ^3^Department of Ethology and Companion Animal Science, Faculty of Agrobiology, Food and Natural Resources, Czech University of Life Sciences Prague, Prague, Czechia

**Keywords:** *Hydatigera*, *Taenia*, *Versteria*, haplotype, liver, molecular epidemiology, small mammals, Czech Republic

## Abstract

**Background:**

Larval stages of carnivore cestodes in rodents have been extensively studied for decades, primarily based on morphological indicators. Numerous datasets, particularly from Central Europe, exist on this topic. Traditionally, the shape, number, and size of hooks have been key distinguishing features. However, recent findings suggest that morphology alone may not provide accurate identification. In this study, rodent livers from various habitats across five regions of the Czech Republic were examined for the presence of taeniid larval cestodes.

**Methods:**

A total of 2,934 rodent specimens were collected using snap traps between 2014 and 2022. Taeniid metacestodes detected in these intermediate hosts were subsequently subjected to species determination through molecular (targeting the cytochrome oxidase subunit I gene) and morphological examinations.

**Results:**

The following cestodes were identified using molecular methods: *Hydatigera kamiyai* (found in *Apodemus flavicollis*, *Apodemus sylvaticus*, *Microtus arvalis*, and *Microtus agrestis*), *Taenia martis* (in *A. flavicollis*, *M. arvalis*, and *Myodes glareolus*), and *Versteria mustelae* (in *M. arvalis* and *My*. *glareolus*). Sequencing of 55 PCR-positive isolates revealed 13 haplotypes of *H*. *kamiyai*, one of *T*. *martis* and four of *V*. *mustelae*. Further, we also identified co-infection by more metacestode species or haplotypes. No rodent liver samples tested were positive for *Echinococcus multilocularis* or *Hydatigera taeniaeformis* sensu stricto, either through morphological or molecular identification methods. Furthermore, no other cryptic species were detected during this study.

**Conclusion:**

This study presents the first comprehensive molecular data on the aforementioned zoonotic cestode species in the Czech Republic. The findings demonstrate the importance of using molecular techniques for Taeniidae species determination, as morphological methods may lead to erroneous classifications. Furthermore, this research highlights the importance of accurate diagnostic techniques, enabling the development of effective prevention and control strategies by verifying host–parasite relationships.

## 1 Introduction

The cestode family Taeniidae comprises approximately 50 endoparasitic species across four genera: *Echinococcus* Rudolphi, 1801; *Hydatigera* Lamarck, 1816; *Taenia* Linnaeus, 1758; and *Versteria* Gmelin, 1790. Members of this parasite group have an indirect life cycle, with herbivores and insectivores primarily serving as intermediate hosts (IH), while carnivores act as definitive hosts (DH). Metacestodes, the larval stages of tapeworms, can infect a wide range of host species of veterinary and/or public health importance, potentially resulting in significant economic losses (1, 2).

Rodents (Rodentia) frequently serve as IH for taeniid cestodes (1–3). As the most abundant and successful group of mammals (Mammal Diversity Database), rodents possess a wide capacity to spread various pathogens, making them ideal indicators for epidemiological studies. Numerous researchers have investigated the occurrence of cestode larval stages in rodents. However, most of these studies have relied solely on morphological identification (4–9), while investigations employing molecular-level determinations remain limited (3, 10–19).

In this context, rodents can serve as important indicators of the prevalence of dangerous tapeworms affecting humans. It should be emphasized that while many cestode genera contain zoonotic species, *Echinococcus multilocularis* is particularly noteworthy as it causes one of the deadliest parasitic diseases in humans, proving fatal if left untreated (20). In Europe, dozens of cases are diagnosed annually, with nine cases reported in the Czech Republic alone in 2023 (National Reference Centre for Epidemiological Data Analysis, State Institute of Health). The prevalence of *E*. *multilocularis* in rodents varies according to numerous factors, and the roles of IH have been studied across various European countries (21). However, this topic still requires attention due to limited data, especially from the Czech Republic. Moreover, other tapeworms involving rodents in their life cycles, including *Taenia* or *Versteria* species, can also pose risks to humans (22).

Despite the medical relevance of rodent-borne helminths, knowledge of the extraintestinal helminth fauna in rodents from the Czech Republic remains limited. Previous epidemiological studies have documented several cestode species (with mammalian DHs) in Czech rodents, including: *Taenia pisiformis*, *T*. *crassiceps*, *T*. *polyacantha*, *T*. *martis*, *Hydatigera taeniaeformis* sensu lato, *Versteria mustelae*, and *Mesocestoides lineatus* (4, 6, 23–27).

To our knowledge, comprehensive data on the prevalence and molecular identification of taeniid metacestodes in Czech rodents, including genetic variation determination, are entirely lacking. The recent reclassification of *H*. *taeniaeformis* s.l. into *H*. *taeniaeformis* sensu stricto, *H*. *kamiyai*, and *Hydatigera* sp. have highlighted the need for more focused research on these species worldwide (11). Particularly, there is a need to verify the occurrence of *H*. *taeniaeformis* s.s. and *H*. *kamiyai* strobilocerci in the Central European region and identify their haplotypes.

In the present study, we sought to identify metacestodes in rodents captured between 2014 and 2022. It was aimed to provide proper identification supplemented by genetic variation descriptions, addressing the current lack of data from the studied region.

## 2 Materials and methods

### 2.1 Sample locations

**Figure 1:**
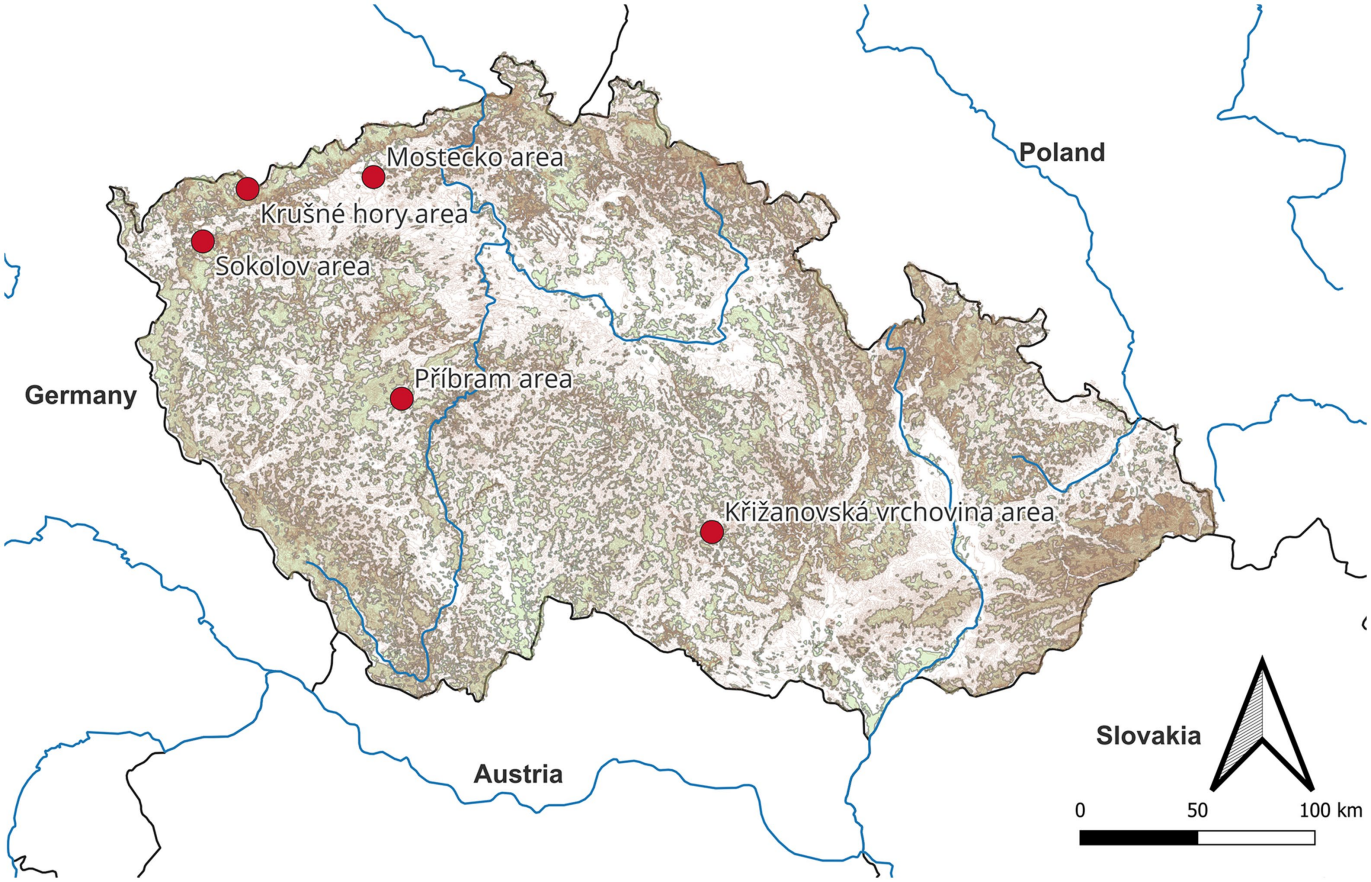
Map of localities investigated. Forest covers are represented in green and mountains are presented in brown.

The study was conducted in five regions of the Czech Republic: Sokolov area, Mostecko area, Krušné hory area, Příbram area, and Křižanovská vrchovina area. The Sokolov area is a reclaimed post-coal mining landscape, featuring mowed meadows, pastures (clover-grass mixtures), fields, and occasional forests (various coniferous and deciduous tree species) and wetlands. Samples were collected between 2014 and 2022. The Mostecko area is also a reclaimed post-coal mining landscape, including grasslands, pastures, and fields. Sampling was obtained from 2017 to 2020. In the Příbram area, sites were in the vicinity of ore mines, comprising grasslands and meadows. Samples were collected between 2014 and 2016. The Krušné hory area is predominantly forested, featuring blue spruce, larch, and occasional rowan. Sampling took place in 2019. The Křižanovská vrchovina area is also predominantly forested and dominated by spruce and beech. Samples were collected in 2022 (Figure 1).

### 2.2 Sample collection

A total of 2,934 rodent specimens were collected using snap traps across all study sites. Five, 25 and 70 localities were repeatedly monitored in Příbram, Mostecko and Sokolov areas, respectively. Based on the characteristics of each trapping plot, traps were established in 2 lines at a distance of 25 m between the lines with 5 m intervals between traps or in quadrates with 5 m grid. The number of traps per site ranged from 25 to 81 according to the habitat size monitored. Eight localities were monitored during 2019 in the Krušné hory area. Animals were sampled on 8 quadrate grids (10 × 10 traps, span 10 m) and along 10 trap-lines (50 trapping points, span 5 m). The quadrate grids and trap lines were situated in deforested habitats with dense growth of the reed-grass and a new plantation of tree species. Six localities were monitored during spring 2022 in the Křižanovská vrchovina area. The trapping plots were in three habitat types: the Norway spruce forest, the mixed forest with a dominance of European beech, and permanent grassland (meadow), two plots each. The 40 traps were set up with 10 m spacing (4 × 10 traps) at each quadrate/site.

Small terrestrial rodents were trapped using simple snap traps baited with cotton candlewicks soaked with mixture of plant oil, flour and rendered bacon. The trapped specimens were processed according to standard mammalogical techniques comprising species, sex, age determination, and basic morphological measurements (body weight, tail length, hind foot, and ear).

All experimental procedures were conducted in compliance with Czech legislation (section 29 of Act No. 246/1992 Coll. on the protection of animals against cruelty, as amended by Act No. 77/2004 Coll.).

### 2.3 Parasitological examination

Each captured individual was subjected to necropsy, with a particular focus on the liver. In addition to macroscopic examination, livers were held over and palpated to search for parasitic lesions within the liver parenchyma. Any visible cysts, spots, lesions, or other abnormal formations on the livers were isolated for further analysis. The initial selection of worms was based on the characteristic scolex morphology of Taeniidae. Fully developed cestode larval stages were morphologically identified to species level based on cyst shape, size, and location in the host as well as on the size, number, and shape of rostellar hooks (3, 7, 11). In either case, all worms were analyzed individually. Cysts and any visible spots or lesions were preserved in 70% ethanol until further examination. Small pieces of the host’s infected tissue (cysts/lesions) were used for molecular identification.

### 2.4 Molecular analysis

Individual specimens (fixed in 70% (v/v) ethanol or thawed material) were transferred to Eppendorf tubes for DNA extraction. Genomic DNA (*n* = 89) was extracted using the NucleoSpin tissue XS kit (Macherey-Nagel, Düren, Germany), following the manufacturer’s instructions. DNA was stored at −20°C until use in polymerase chain reaction (PCR) assays targeting the mitochondrial *cox1* gene, selected for characterization of species and haplotypes. PCR amplifications were performed in reaction mixtures consisting of 12.5 μL of GoTaq® G2 Hot Start Green Master Mix (Promega, Madison, WI, United States), 0.4 μM of each primer, and 5 μL DNA template. PCRs were carried out using primers JB3 (forward): 5′-TTTTTTGGGCATCCTGAGGTTTAT-3′ and JB4.5 (reverse): 5′-TAAAGAAAGAACATAATGAAAATG-3′ (28), to amplify a part of the gene under the following conditions: 1 cycle of 3 min at 95°C as an initial hot start step, followed by 36 cycles of 30 s at 94°C, 45 s at 55°C, 30 s at 72°C, and a final extension step at 72°C for 10 min. A negative control using RNase/DNase-free water was included in each reaction. PCR products were assessed by gel electrophoresis on a 1% (w/v) agarose gel and purified using the ExoSAP-IT™ Express PCR Product Cleanup Reagent Kit (Thermo Fisher Scientific), as per the manufacturer’s protocol. Purified amplicons were sequenced by Eurofins Genomics (Ebersberg, Germany) using forward and reverse primers. Nucleotide sequences derived in this study have been deposited in GenBank (accession numbers: PQ868574; PQ868575; PQ868896; PQ868998; PQ869002; PQ869008; PQ869162; PQ869198; PQ869199; PQ869203; PQ869225; PQ869280; PQ869284; PQ869303; PQ869304; PQ870821; PQ870820; PQ870818; PQ870819; PQ870824; PQ870826; PQ870817; PQ870825; PQ870822; PQ870823; PQ870827). Sequences were bidirectionally manually edited using FinchTV software (Geospiza Inc., Seattle, WA, United States) and compared using the NCBI Basic Local Alignment Search Tool (BLASTn)^1^. Alignment of sequences was performed using an online version of MAFFT Alignment v7 (29). Phylogenetic relationships of obtained nucleotide sequences were analyzed using the MEGA11 software (30). Phylogenetic relationships were reconstructed using the maximum likelihood (ML) method, based on the Hasegawa-Kishino-Yano parameter model (31) with a gamma distribution rate and a proportion of invariant sites (HKY + G + I), bootstrapped at 1,000 replicates. Sequences of *Echinococcus granulosus* sensu lato (GenBank accession number: MH300987) and *E*. *multilocularis* (GenBank accession number: OR911432) were used as outgroups.

## 3 Results

### 3.1 Morphological assessment

A total of 2,934 individuals representing 9 rodent species were captured and examined by necropsy. Cysts, visible spots, or lesions were isolated from the livers of 89 rodents. Among these abnormalities, 55 were confirmed as cestode infections through DNA sequencing and, in some cases, by morphological features. Only the cysts or lesions that were molecularly identified to the species level were used for morphological description of the larvae. These cysts or macroscopically visible spots or lesions were found in only five out of nine examined rodent species (listed in Table 1).

**Table 1:** List of rodent specimens obtained using snap traps and examined during the study period 2014–2022.

Host species	Common name	Total specimens	No. of samples*	No of cysts/lesions**
Yellow-necked field mouse	*Apodemus flavicollis* (Melchior, 1834)	533	21	16/5
Ural field mouse	*Apodemus uralensis* (Pallas, 1811)	7	0	0
Wood mouse	*Apodemus sylvaticus* (Linnaeus, 1758)	517	5	5/0
Eurasian harvest mouse	*Micromys minutus* (Pallas, 1771)	20	0	0
House mouse	*Mus musculus* (Linnaeus, 1758)	5	0	0
European water vole	*Arvicola amphibius* (Linnaeus, 1758)	9	0	0
Short-tailed field vole	*Microtus agrestis* (Linnaeus, 1761)	62	1	1
Common vole	*Microtus arvalis* (Pallas, 1778)	1,532	56	51/5
Bank vole	*Myodes glareolus* (Schreber, 1780)	251	6	3/3
	SUM	2,934	89	76/13

*samples = isolate of cyst, spots or lesion at livers used for PCR.

** lesions = isolate of spots or lesion at livers used for PCR.

Larval cestodes were found in only 5 species: *A. flavicollis*, *A. sylvaticus*, *M. arvalis*, *M. agrestis*, and *My*. *glareolus*. No metacestodes were detected in *A. uralensis*, *A. amphibius*, *Micromys minutus*, or *Mus musculus* (*Mu*. *m*. *domesticus* and *Mu*. *m*. *musculus*). Among 55 cestodes the following species were identified: *Hydatigera kamiyai*, *Versteria mustelae* and *Taenia martis*. The prevalence of individual rodent species is given in Table 2; the occurrence of metacestode species in individual areas is shown in Table 3.

**Table 2:** The prevalence of the infection (%) of metacestodes in rodent species from five areas of the Czech Republic.

Host species	Parasite species	No. of rodent specimens	No. of metacestode species*	%
*Microtus arvalis*	*Hydatigera kamiyai*	1,532	32	2.09
	*Versteria mustelae*	1,532	2	0.13
	*Taenia martis*	1,532	1	0.07
*Microtus agrestis*	*Hydatigera kamiyai*	62	1	1.61
	*Versteria mustelae*	62	0	0
	*Taenia martis*	62	0	0
*Apodemus flavicollis*	*Hydatigera kamiyai*	533	8	1.50
	*Versteria mustelae*	533	0	0.19
	*Taenia martis*	533	1	0.19
*Apodemus sylvaticus*	*Hydatigera kamiyai*	517	2	0.39
	*Versteria mustelae*	517	0	0
	*Taenia martis*	517	0	0
*Myodes glareolus*	*Hydatigera kamiyai*	251	0	0
	*Versteria mustelae*	251	2	0.40
	*Taenia martis*	251	1	0.40
	SUM	2,934	50	

* the number of rodent specimens with the molecularly identified taeniid species infection.

**Table 3:** The prevalence of the infection (%) of taeniid species in rodents from five areas of the Czech Republic.

Locality	No. of rodent specimens	No. of infected rodents*	%	No. of specimens with metacestodes					
				*Hydatigera kamiyai*		*Versteria mustelae*		*Taenia martis*	
				*N*	%	*N*	%	*N*	%
Mostecko area	381	18	4.72	16	4.2	1	0.26	1	0.26
Sokolov area	2052	18	0.88	14	0.68	2	0.1	2	0.1
Příbram area	389	8	2.06	8	2.06	0	0	0	0
Krušné hory area	89	4	4.49	4	3.37	0	0	0	0
Křižanovská vrchovina area	23	2	8.70	1	4.35	1	4.35	0	0

* the number of rodent specimens with the molecularly identified taeniid species infection.

Six animals were infected by more than one developmental stage of the cestode parasite, including co-infections involving morphologically distinct parasite species in two cases. *Microtus arvalis* exhibited the highest number of cysts per host, with 2–4 cysts or strobilocerci found per individual (in Sokolov and Mostecko areas). The second most prevalent species was *A. flavicollis*, with up to three cysts/strobilocerci per animal (in Krušné hory and Mostecko areas). Lesions and cysts morphologically resembling any metacestodes were subjected to molecular identification.

**Figure 2:**
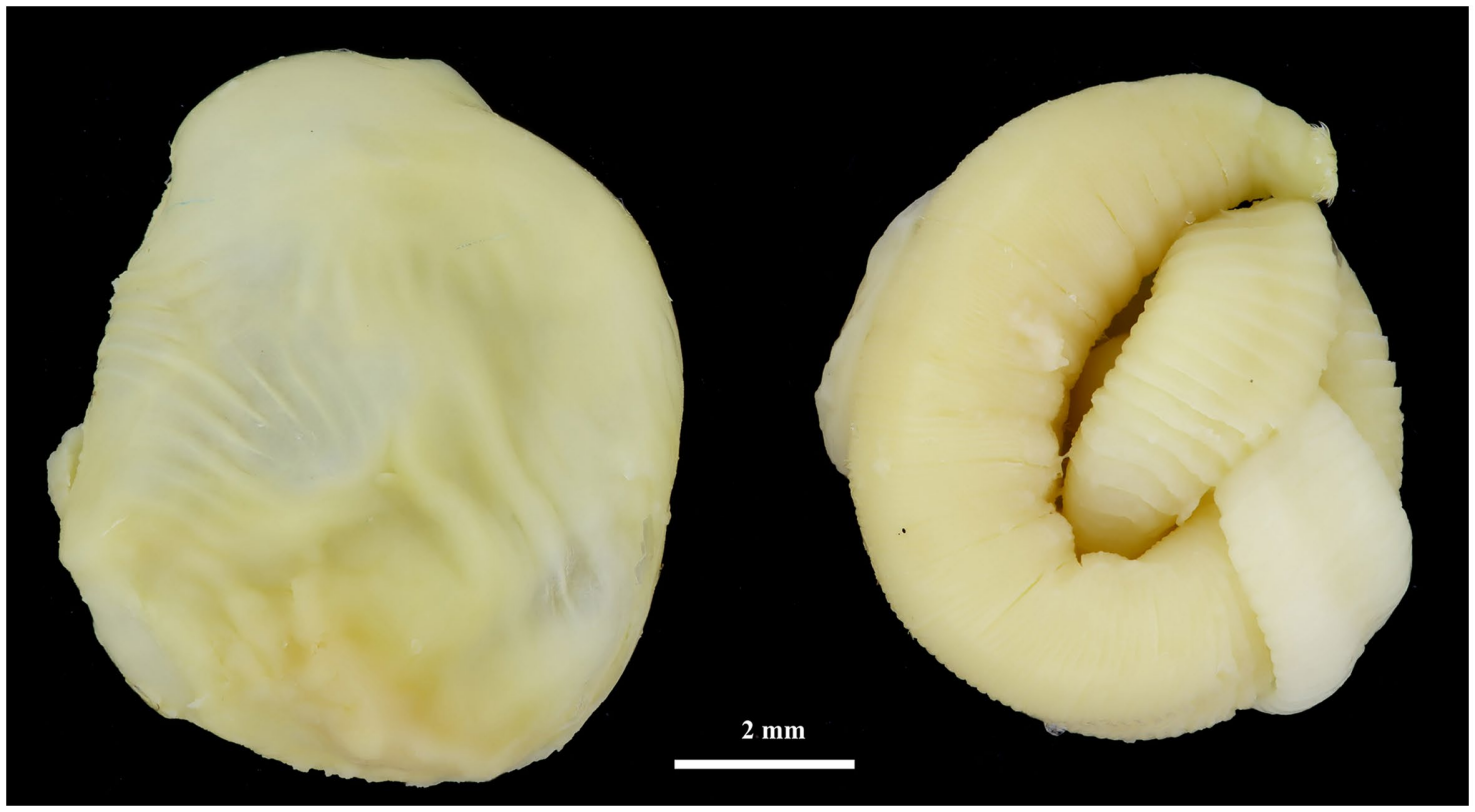
Cyst (left) and strobilocercus (right) of *H*. *kamiyai* removed from the liver of *M. arvalis* (Mostecko area).

**Figure 3:**
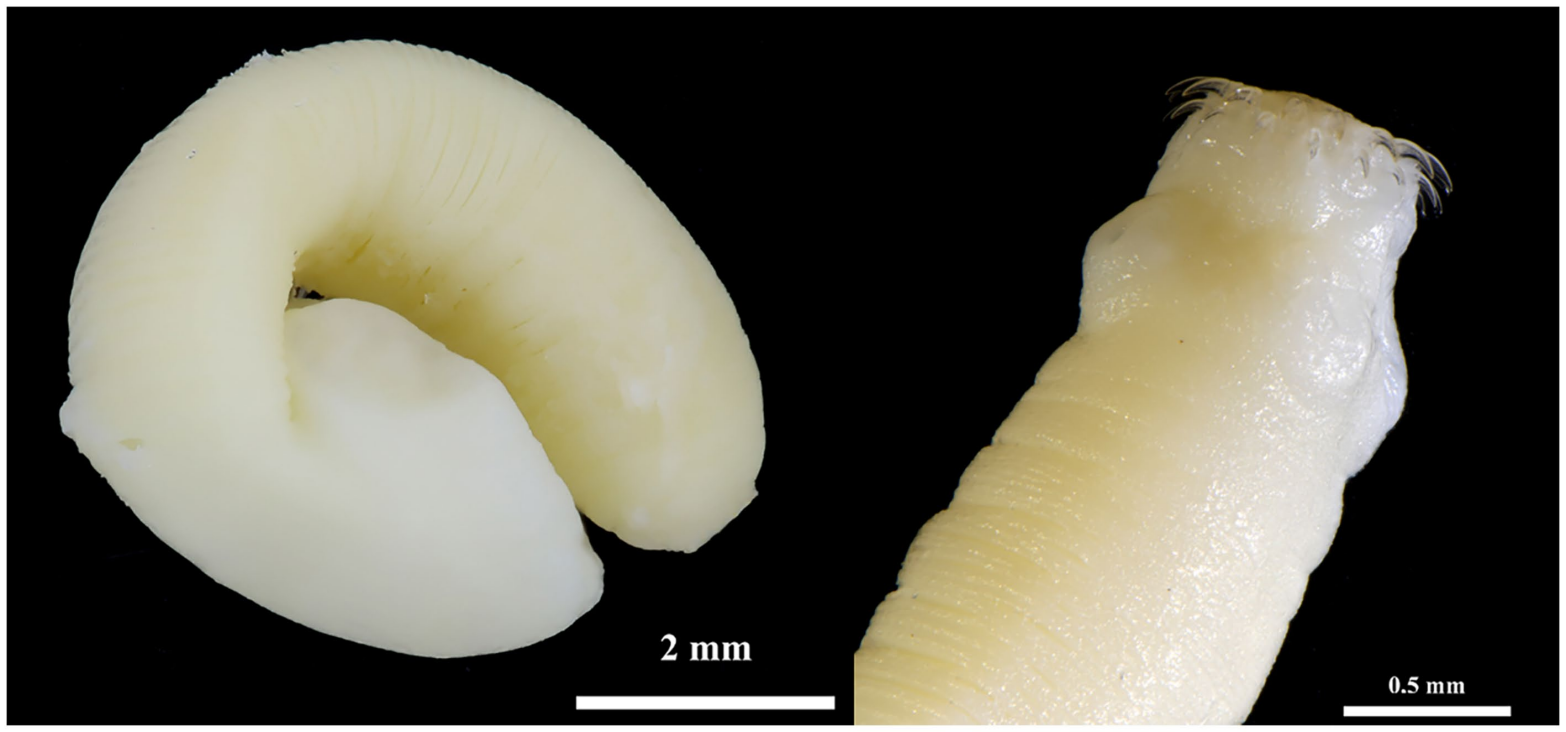
*Hydatigera kamiyai*. Strobilocercus (left) and detail of the scolex of another individual (right) obtained from two specimens of *M. arvalis* (Příbram area).

*Hydatigera kamiyai* Iwaki, 2016 (larval stages, Figures 2, 3) was identified in 43 rodents from the Sokolov, Mostecko, Příbram, and Krušné hory areas. The hosts included *A. flavicollis*, *A. sylvaticus*, *M. arvalis*, and *M. agrestis*, with the liver serving as the primary predilection site. The infection intensity ranged from 1 to 4 cysts. Morphological examination revealed ovular or globular cysts, whitish to yellow, measuring 3.24–15.14 mm on the liver. These cysts contained white pseudosegmented strobilocerci with terminal bladders, with individual strobilocerci varying in length from 6.27–7.02 mm. Scolices with hooks were observed only in cysts with a diameter of at least 0.5 mm. The scolex diameter ranged from 1.1–1.3 mm (without suckers), with an average of 1.2 mm, and featured 4 suckers measuring 0.331–0.497 mm in diameter. The prominent suckers ranged in height from 60 to 198 μm. Two hook crowns were on the scolex, with a crown diameter of 0.792–1.016 mm. The total hook count varied from 26 to 36, comprising 11–18 large hooks and 14–18 small hooks. Large hooks measured 352–466 μm, while small hooks ranged from 245 to 268 μm. Detailed hook measurements are provided in Table 4.

**Table 4:** Various large and small hook dimensions of metacestodes of *H*. *kamiyai*.

		Large hooks (μm)			Small hooks (μm)		
	*n*	mean	min	max	mean	min	max
Total Length (TL)	20	425	352	466	258	245	269
Total Width (TW)	20	183	145	199	127	111	145
Basal Length (BL)	20	272	26	319	151	136	180
Apical Length (AL)	20	201	178	214	143	133	156
Guard Length (GL)	20	90	69	109	61	38	80
Guard Width (GW)	20	71	57	91	47	38	56
Blade Curvature (BC)	20	43	32	65	32	14	45
Handle Width (HW)	20	64	45	87	36	28	52

**Figure 4:**
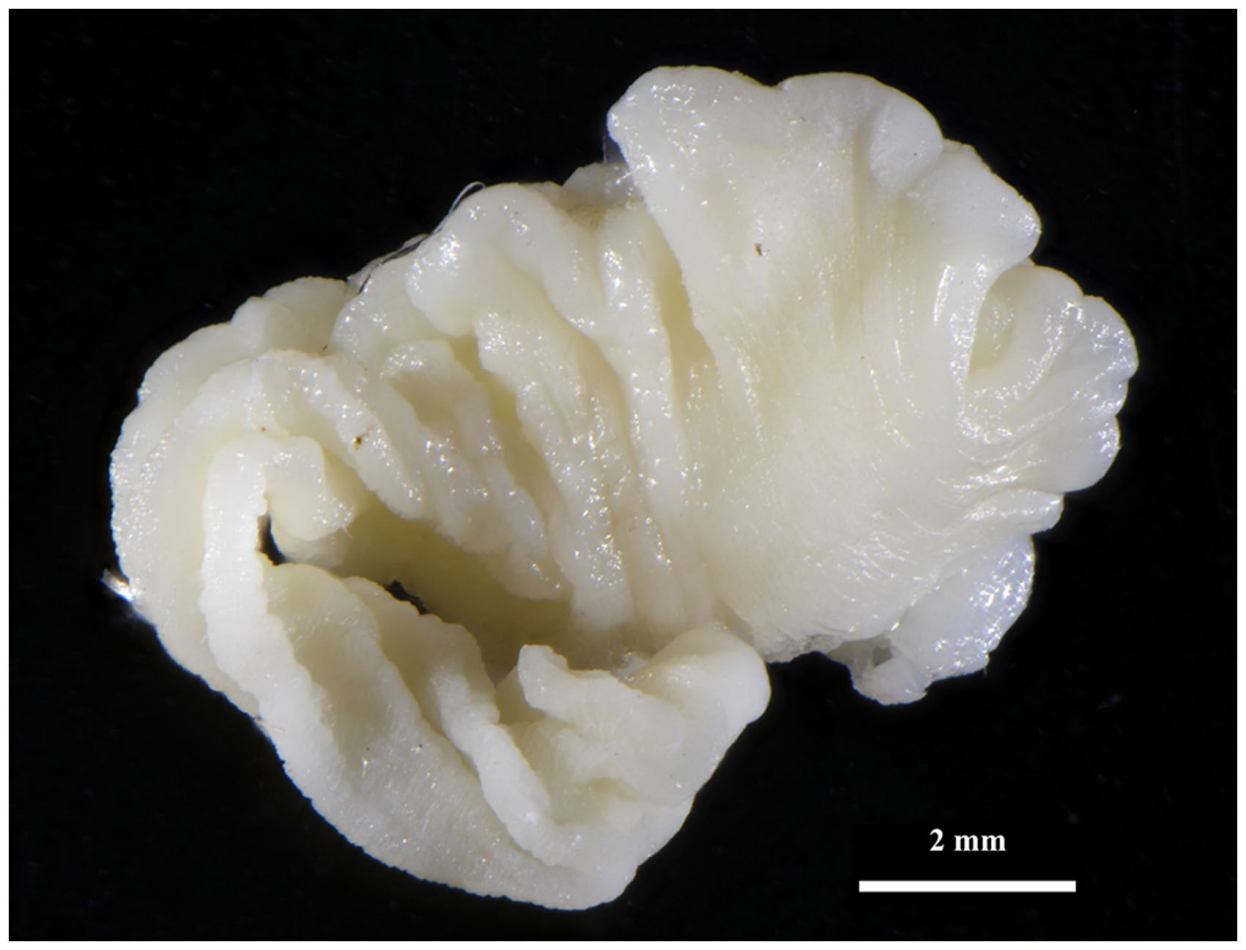
Strobilocercus of *T*. *martis* removed from liver of *My*. *glareolus* (Sokolov area).

*Taenia martis* (Zeder, 1803) larval stages (Figure 4) were also identified in three rodents in the Sokolov and Mostecko areas. The hosts included *A. flavicollis*, *M. arvalis*, and *My*. *glareolus*, with the larvae found on the liver. The infection intensity ranged from 1 to 3 larval stages per host. The pseudosegmented metacestodes measured 2.9–10.1 mm in length and 2–4.2 mm in width, with some specimens exhibiting a slender tail-like structure at the posterior end. Despite careful examination, no scolices or hooks were observed in these larval stages.

*Versteria mustelae* (Gmelin, 1970) (syn. *Taenia tenuicollis* Rudolphi, 1919) larval stages were identified in the Sokolov, Mostecko, and Křižanovská vrchovina areas. The hosts included *M. arvalis* (two animals) and *My*. *glareolus* (two animals), with the liver serving as the primary site of infection. The infection intensity was consistently one metacestode per host. It’s worth noting that Nakao et al. (2) proposed the creation of a new genus, *Versteria*, for *Taenia mustelae*. In all cases, only spots and lesions measuring 1 to 2 mm were observed in the liver. No scolices or hooks were found during the examination of these lesions; the identification of this species was carried out using molecular methods.

### 3.2 Molecular identification

In this work, we detected the following cestodes using molecular methods: *H*. *kamiyai*, *T*. *martis*, and *V*. *mustelae*. Among the 89 DNA isolates from cysts, spots, and lesions, 55 sequences were successfully amplified for the partial *cox1* gene. These comprised 13 haplotypes of *H*. *kamiyai*, one of *T*. *martis*, and four of *V*. *mustelae* (Table 5). No PCR product was obtained from the remaining isolates.

**Table 5:** Metacestodes characterization with haplotype identification supplemented with the degree of similarity among data with other sequences retrieved from GenBank.

Parasite species	Haplotype	Isolate codes (GenBank acc. no.)	Host species (no. of similar obtained seq.)	Genetic similarity at *cox1* gene (host species; geographic origin; GenBank acc. no.)
*Hydatigera kamiyai*	HkCZ1*	MarCZ1 (PQ868574)	*M. arvalis* (4)	99.49% (*F. catus*; Finland; EU861478) 2 nucleotides A/G (282 bp) and C/T (330 bp)
	HkCZ2	AflCZ2 (PQ868575) MarCZ2 (PQ870817)	*A. flavicollis* (1) *M. arvalis* (5)	100% (*A. flavicollis*; Bosnia and Herzegovina; KT693077)
	HkCZ3	AflCZ3 (PQ868896) AsyCZ3 (PQ870819) MagCZ3 (PQ870818) MarCZ3 (PQ870820)	*A. flavicollis* (2) *A. sylvaticus* (1) *M. agrestis* (1) *M. arvalis* (7)	100% (*F. catus*; Finland; EU861478)
	HkCZ4*	MarCZ4 (PQ868998)	*M. arvalis* (1)	99.75% (*F. catus*; Poland; KF702312) 1 nucleotide G/A (120 bp)
	HkCZ5*	MarCZ5 (PQ869002)	*M. arvalis* (2)	99.75% (*F. catus*; Finland; EU861478) 1 nucleotide C/T (42 bp)
	HkCZ6	MarCZ6 (PQ869008)	*M. arvalis* (5)	100% (*F. silvestris catus*; Finland; KT693081)
	HkCZ7	AflCZ7 (PQ870821) MarCZ7 (PQ870826)	*A. flavicollis* (3) *M. arvalis* (4)	100% (*F. catus*; Poland; KF702312)
	HkCZ8*	MarCZ8 (PQ869162)	*M. arvalis* (4)	99.75% (*F. silvestris catus*; Finland; KT693081) 1 nucleotide
	HkCZ9	AsyCZ9 (PQ870822) MarCZ9 (PQ870823)	*A. sylvaticus* (1) *M. arvalis* (3)	100% (*F. catus*; Estonia; MT407624)
	HkCZ10*	AflCZ10 (PQ869198)	*A. flavicollis* (1)	99.75% (*F. catus*; Finland; EU861478) 1 nucleotide A/G (171 bp)
	HkCZ11*	MarCZ11 (PQ869199)	*M. arvalis* (1)	99.49% (*F. catus*; Finland; EU861478) 2 nucleotides C/T (42 bp and 370 bp)
	HkCZ12*	MarCZ12 (PQ869203)	*M. arvalis* (1)	99.75% (*F. catus*; Finland; EU861478) 1 nucleotide T/C (257 bp)
	HkCZ13*	AflCZ13 (PQ869225)	*A. flavicollis* (1)	99.75% (*F. catus*; Poland; KF702312) 1 nucleotide C/T (135 bp)
*Taenia martis*	TmCZ1	AflCZ14 (PQ870824) MarCZ14 (PQ870825) MglCZ14 (PQ870827)	*A. flavicollis* (1) *M. arvalis* (1) *My*. *glareolus* (1)	100% (*My*. *glareolus*; Denmark; EU544553)
*Versteria mustelae*	VmCZ1	MarCZ15 (PQ869280)**	*M. arvalis* (1)	100% (*My*. *glareolus*; Finland; EU544559)
	VmCZ2	MarCZ16 (PQ869284)**	*M. arvalis* (1)	99.74% (*My*. *glareolus*; Finland; EU544559) 1 nucleotide G/A (288 bp)
	VmCZ3*	MglCZ17 (PQ869304)	*My*. *glareolus* (1)	99.24% (*My*. *glareolus*; Finland; EU544559) 3 nucleotides A/G (288 bp and 337 bp) and C/T (354 bp)
	VmCZ4*	MglCZ18 (PQ869303)	*My*. *glareolus* (1)	99.49% (*My*. *glareolus*; Finland; EU544559) 2 nucleotides G/A (84 bp) and A/G (282 bp)

*new haplotype according to SNP within 396 bp.

**short sequence MarCZ15 (345 bp) and MarCZ16 (386 bp), all other 396 bp.

**Figure 5:**
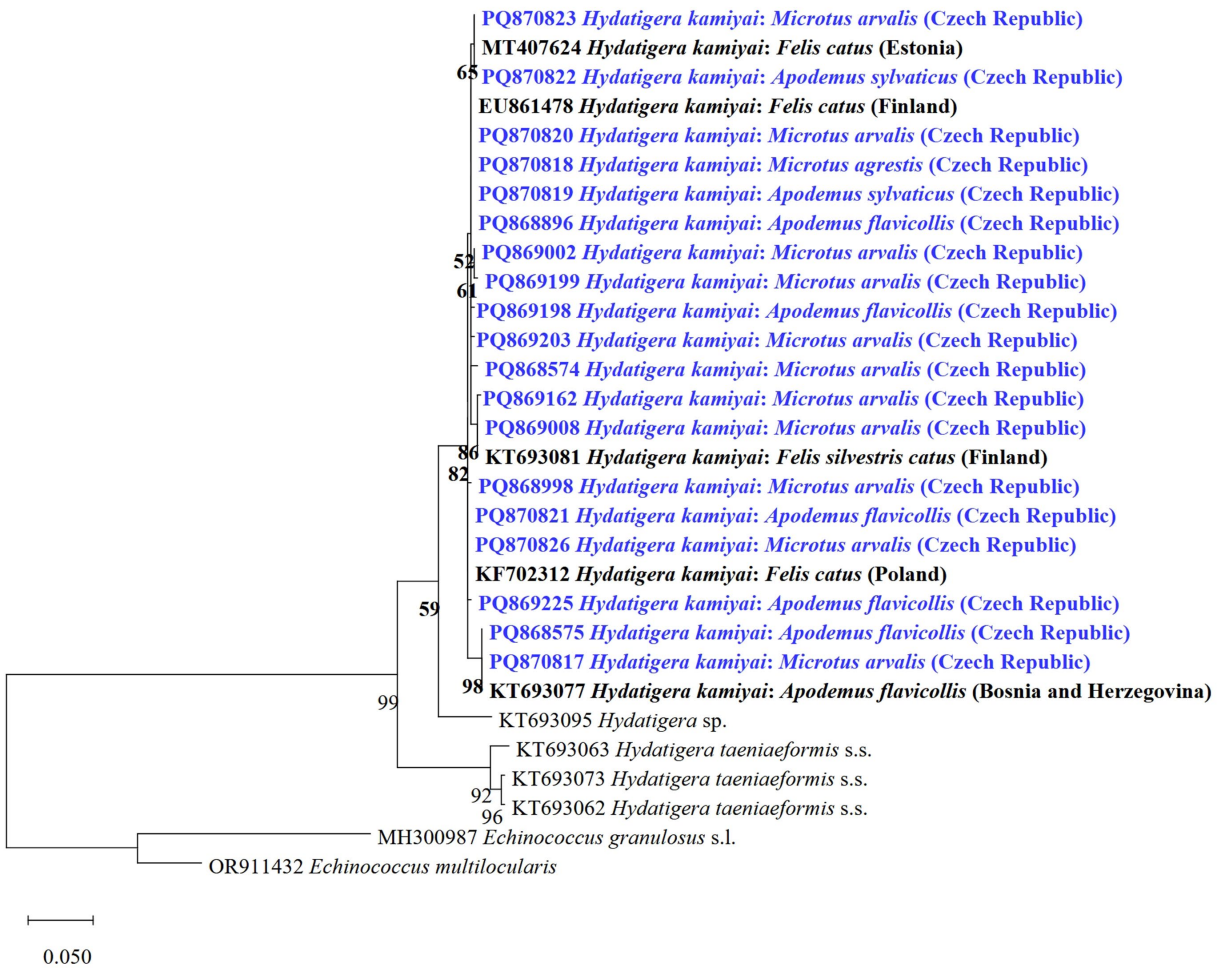
Maximum likelihood tree based on *cox1* gene for representatives of *H*. *kamiyai* and two related species of *Hydatigera* from various hosts and countries. Sequences obtained in the present study are shown in blue. Sequences of *E. granulosus* s.l. and *E*. *multilocularis* were used as outgroups.

*Hydatigera kamiyai* was the most frequent finding, with 48 sequences obtained from 4 host species comprising 13 haplotypes (HkCZ1-HkCZ13). These haplotypes differed by single nucleotide polymorphisms (SNPs). Five haplotypes were 100% identical to published *cox1* sequences of *H*. *kamiyai* described from small mammal hosts, for example, *A. flavicollis* from Bosnia and Herzegovina (GenBank no. KT693077) or *Felis catus* from Estonia, Finland, and Poland (GenBank nos: MT407624, EU861478, KF702312) (see Table 5 for details). The remaining sequenced isolates differed from each other by 1–2 SNPs (99.75–99.49% identity). The phylogenetic tree based on *cox1* for representatives of *H*. *kamiyai* (Figure 5) showed that sequences obtained in the present study are grouped in a single clade with the same species from other hosts and countries. The subclades within this single clade are formed by species sharing the same haplotypes (e.g., sequences PQ868575, PQ870817 and KT693077 shared haplotype) or new haplotypes.

Five animals were positive for multiple *H*. *kamiyai* strobilocerci. In two *A. flavicollis* and three *M. arvalis*, different haplotypes were identified within the same host. Co-infection of one *M. arvalis* by two cestode species, *H*. *kamiyai* and *T*. *martis*, was identified in one case. Notably, *H*. *taeniaeformis* s.s. and no other cryptic species were found in this study.

**Figure 6:**
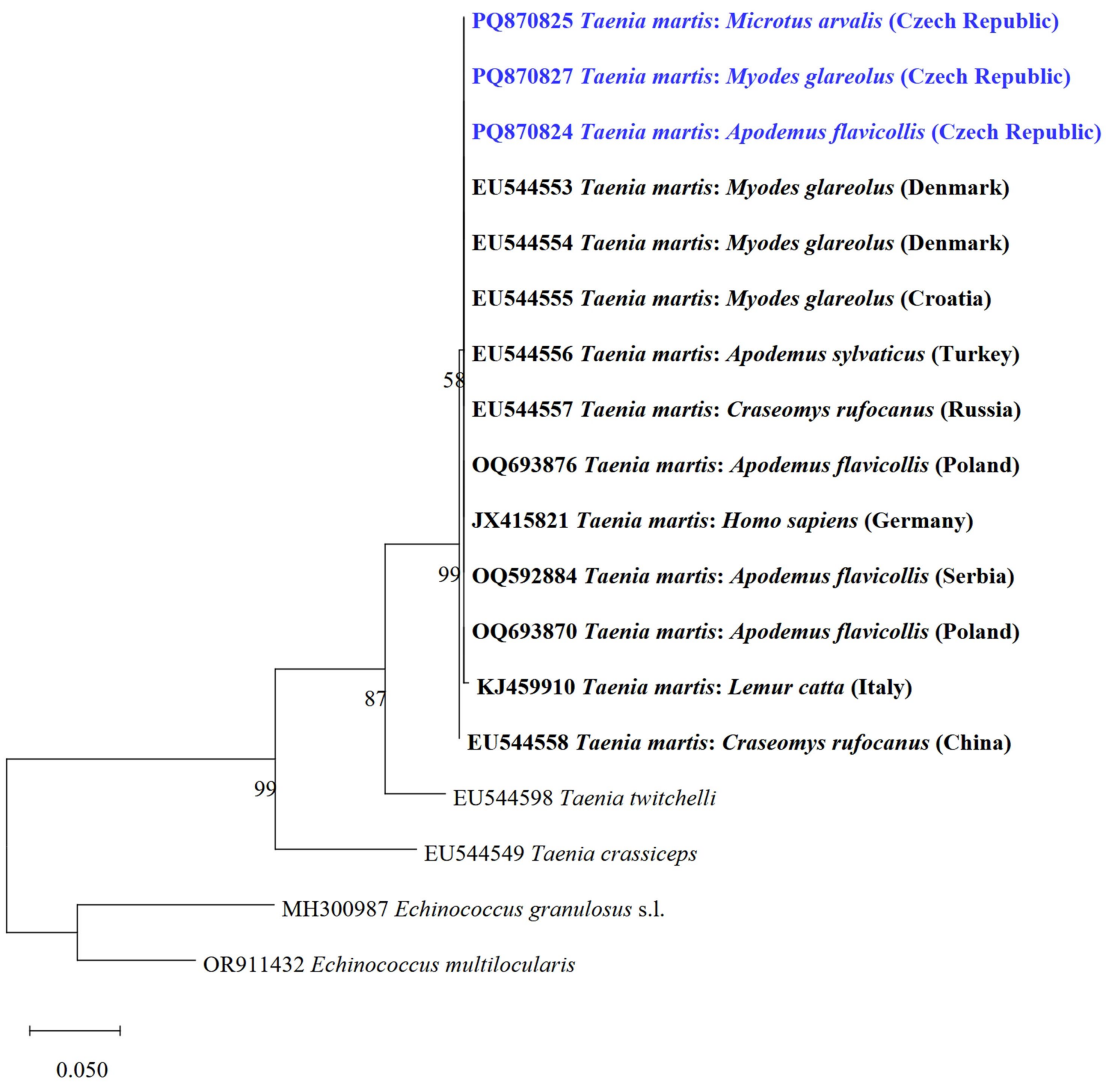
Phylogenetic tree of the *cox1* gene of *T*. *martis* and two related species of *Taenia* from various host species and countries using the maximum likelihood method. Sequences of *E. granulosus* s.l. and *E*. *multilocularis* were used as outgroups. Sequences obtained in this study are marked in blue.

The *T*. *martis* metacestode was molecularly determined for the first time in rodents from Central Europe. We detected metacestodes of *T*. *martis* using *cox1* gene PCR in *M. arvalis*, *My*. *glareolus*, and *A. flavicollis*. All three isolate sequences (TmCZ1 haplotype) were 100% identical to *My*. *glareolus* from Denmark (GenBank no.: EU544553). Resulted phylogenetic tree based on *cox1* for representatives of *T*. *martis* (Figure 6) showed that sequences obtained in the present study are grouped in a clade with the same species from other hosts and countries.

**Figure 7:**
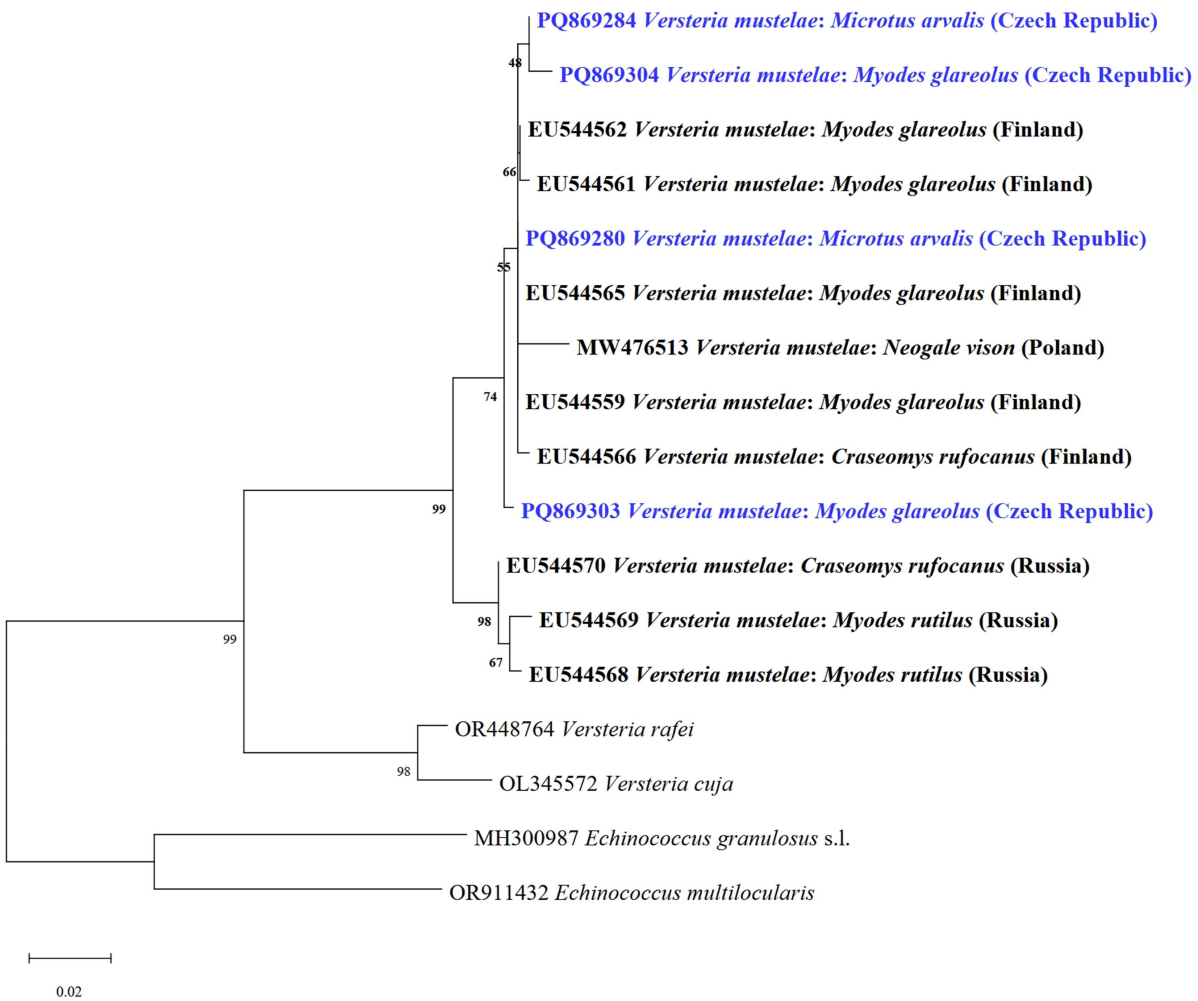
Phylogenetic tree of *V*. *mustelae* and two related species of *Versteria* from various hosts and countries based on of the *cox1* gene using the maximum likelihood method. Sequences of *E. granulosus* s.l. and *E*. *multilocularis* were used as outgroups. Sequences obtained in this study are marked in blue.

We also identified DNA of *V*. *mustelae* metacestodes (VmCZ1-VmCZ4) in four voles solely through molecular analyses, detecting them twice in *M. arvalis* and twice in *My*. *glareolus*. This included two newly identified haplotypes. Our sequences showed 99.24–100% similarity to a haplotype from Finland (GenBank no. EU544559). This represents the first molecular finding of *V*. *mustelae* in Central Europe. The phylogenetic tree analysis based on *cox1* revealed that our isolates grouped with *V*. *mustelae* isolates (Figure 7). All other lesions or cysts isolated for molecular determination were negative for *E*. *multilocularis*.

## 4 Discussion

Due to a lack of data concerning the current prevalence, species diversity, and genetic variability of rodent-borne metacestodes in Central Europe, the present study aimed to address these gaps. Previous studies on the presence of taeniid metacestodes lacked molecular information, making this study the first to provide valuable data on the molecular identity of some species. All species reported herein were previously known (4, 7, 26, 27, 32), although all species (*H*. *kamiyai, T*. *martis* and *V*. *mustelae*) represent new geographical records in Central Europe.

We found a maximum of four metacestodes in a single host. All co-infections of one individual by one or more larvae were identified individually for the first time, highlighting the need for further studies on molecular diversity from individual stages in whole animals, similar to research on other tapeworm species such as *Echinococcus granulosus* s.s. (33). Multiple parasite stages in one animal are commonly recorded; for example, *T*. *martis* has been found in multiple infections. Murai (32) reported five cysts per animal, Schmidt (9) eight cysts, Prokopič and Mahnert (26) up to 12 cysts, and Frank and Zeyhkle (34) even detected 43 cysts of *T*. *martis* in one common muskrat (*Ondatra zibethicus*). Similarly, *V*. *mustelae* is often detected as a multi-infection, most often up to 10 cysts per animal (9, 35). Prokopič and Mahnert (26) found 18 cysts per animal, while Murai (32) found up to 20. Unfortunately, none of these previous results were supported by molecular analyses and characterization of all metacestodes, as was done in the present study.

Our study also includes a morphological description of *H*. *kamiyai* metacestodes, which were determined using the *cox1* gene. *Hydatigera kamiyai* and *H*. *taeniaeformis* s.s. differ molecularly, though less distinctly, and most features overlap (11). However, these authors showed that both congeners differed significantly in seven large and small hooks characters. Unlike molecular analyses, no other morphological studies of these species have been conducted to date, unlike molecular analyses (18).

The present study revealed a slight difference in the morphology of the large hooks of *H*. *kamiyai* metacestodes compared to those described by Lavikainen et al. (11), which measured 421–461 μm, while our samples ranged from 352 to 466 μm. This variability in hook length may be related to the amount of material or the age of the larvae.

Finally, we found that the hook numbers differed for large and small hooks on most tapeworm larvae specimens measured. This validates molecular methods as important tools that greatly facilitate taxonomic identification of organisms globally. However, the results of measuring the metacestodes of *H*. *kamiyai* in this study indicate that this tapeworm has considerable morphological variability, and molecular identification will be necessary for all future studies.

Additional studies are needed, particularly to elucidate whether *H*. *kamiyai* is also common in cats and other potential domestic or wildlife DH species present in the Czech Republic, as observed in other countries (see Table 1 in reference 11). Apart from Lavikainen et al. (11) *H*. *kamiyai* has been recorded in the following studies: by Bajer et al. (16) in *M. arvalis* and *My*. *glareolus* (Poland), by Martini et al. (17) in *O. zibethicus* (Luxembourg), by Miljeviš et al. (18) in *A. flavicollis*, *A. agrarius*, *M. arvalis* and *Crocidrua leucodon* (Serbia). Outside of Europe, it has been molecularly determined recently only from the Tibetan Plateau from *Neodon fuscus* (19). None of these reports contain morphological characteristics. This implies that the haplotype occurrence and diversity remain unknown, and a study on DHs is necessary to obtain data specific to the Czech Republic. More samples, particularly from murine rodents or DHs, are required to reliably determine the possible occurrence of *H*. *taeniaeformis* s.s. or *Hydatigera* sp. in Central Europe.

Studies on intra-species variations and differences in the distribution of *H*. *kamiyai* are very limited. Its diversity across various IHs in our samples and elsewhere (18) is an interesting topic for future research, similar to studies on *E*. *multilocularis* (36). The present data contain newly described haplotypes alongside those typical for Europe, adding value to other studies focused on haplotype network analysis or predominant variant determination. In future studies, these data could be used for DH identifications. Previously, 22 haplotypes (BB1-B22; 396 bp) were described by Lavikainen et al. (11), with additional haplotypes later reported by other authors (18), collectively supporting a high level of genetic diversity worldwide. The next species we found in the examined rodents was *T*. *martis*, one of the most widely distributed helminths of Mustelidae occurring throughout the Palearctic region in the *Martes* genus. In recent years, metacestodes have been frequently found in various primates and humans (37–39), indicating considerable zoonotic potential for this tapeworm.

The importance of *T*. *martis* is underscored by the fact that martens are common predators in urban and suburban areas (40, 41). Thus, monitoring the occurrence of this tapeworm would be appropriate and reasonable. Its metacestodes have been found in immunocompetent individuals worldwide, including recent cases in Europe. Examples include brain infections reported by Steinsiepe et al. (42) in Switzerland and Eggink et al. (39) in the Netherlands; eye infections documented by Eberwein et al. (43), Koch et al. (44), and Tappe et al. (45) in Germany; and an infection in the pouch of Douglas reported by Mueller et al. (37), also in Germany. Most of these patients lived in rural villages, were involved in agriculture, and reported frequent marten sightings around their homes.

Metacestodes of *T*. *martis* are found in Arvicolinae, Murinae, Cricetinae, Sciurinae, and also in *Myocastor coypus*, *Castor fiber*, and *Sorex araneus* (5, 46–48). The current study is based on molecular identification, and many studies are available for comparison with our molecular data. *Taenia martis* has been further identified by Al-Sabi and Kapel in Denmark (49); Umhang et al. (47) in *Myocastor coypus* in France; Krücken et al. (50) in *My*. *glareolus*, *A. agrarius*, and *A. flavicollis* in Germany; Meyer et al. (51) in pigs in Switzerland; Martini et al. (17) in *O. zibethicus* in Luxembourg; and Miljevič et al. (18) in *A. flavicollis*, *A. sylvaticus*, and *My*. *glareolus* in Serbia. Additionally, Reinhardt et al. (52) used PCR sequencing to identify *T*. *martis* adult tapeworms in raccoons (*Procyon lotor*) from Germany.

*Versteria mustelae*, the third recorded tapeworm species in our study, was molecularly determined for the first time in Central Europe. *Versteria mustelae* is widespread throughout the world; the DHs are mainly mustelids, whereas IHs include many species of Arvicolinae, Murinae, Cricetinae, Sciurinae, as well as *Myocastor coypus*, *Ochotona alpina*, *Eospalax baileyi*, *Talpa europaea*, *S. araneus*, and *Crocidura russula* (5–7, 22, 26, 53–55). Members of the genus *Versteria* have been detected in primates (56) and humans (57). It is commonly reported using morphometric characteristics, especially in voles, and was recently found in *My*. *glareolus* (58–60), *M. agrestis* (61), and *A. amphibius* (62). An experimental study showed limited development of *V*. *mustelae* in laboratory mice (54). Our four identifications of *V*. *mustelae* were based solely on sequencing results, thus adding valuable data for further studies/findings. Only several records worldwide have used molecular methods, such as *V*. *mustelae* determined by Al-Sabi and Kapel in Denmark (49); Al-Sabi et al. (10) in *My*. *glareolus* in Denmark; Umhang et al. (47) in *O. zibethicus* and *Myocastor coypus* in France; Miller et al. (13) in *M. agrestis* and *A. terrestris* in Sweden; Martini et al. (17) in *O. zibethicus* in Luxembourg; and Zhao et al. (63) in *E. baileyi* on the Qinghai-Tibet Plateau.

We further sought to verify the occurrence of *E*. *multilocularis* in rodents, a widespread species among the main DH, red foxes (*Vulpes vulpes*), in the Czech Republic. The pooled prevalence in foxes has long been high, reaching around 25% (e.g., 25.32%; 254 positive/1003 examined red foxes in 2023; Máca, unpublished data), which is considerably higher compared to other European countries (21). However, we found no *E*. *multilocularis* infection among cysts/lesions on the rodents’ livers, similar to findings from Serbia (18). In fact, *E*. *multilocularis* has only been found once in rodents in the Czech Republic, specifically in *My*. *glareolus* (1 positive/36 examined) based on histological examination, but without finding protoscoleces (64). Thus, further studies are needed to fully elucidate the role of rodents in the Czech Republic regarding *E*. *multilocularis* and its distribution in specific areas.

To conclude, we used molecular biology-based methods to show the occurrence of three tapeworms with zoonotic potential in five common rodent species of Central Europe. *Microtus arvalis* was the most frequently trapped rodent species and the most commonly infected with larval cestodes. The metacestodes of *H*. *kamiyai* were also morphologically described and discussed based on molecularly characterized individuals. New findings with described European mitochondrial haplotypes enrich the present study, offering the potential for new research focusing on transmission pathways or variant distribution in broader geographical contexts. Our new data on *T*. *martis* and *V*. *mustelae* represent the only recent records from the Czech Republic supplemented by molecular characterization. These findings can aid in epidemiological studies and advance our understanding of infection transmission pathways in cases of human infections. Future research should focus on urban areas and, regions with high *E*. *multilocularis* prevalence to evaluate the contribution of rodent hosts in spreading this causative agent.

## Data Availability

The datasets presented in this study can be found in online repositories. The names of the repository/repositories and accession number(s) can be found in the article/supplementary material.

## Footnotes

^1^ http://blast.ncbi.nlm.nih.gov/Blast.cgi
